# Parenchymal neuroinflammatory signaling and dural neurogenic inflammation in migraine

**DOI:** 10.1186/s10194-021-01353-0

**Published:** 2021-11-18

**Authors:** Şefik Evren Erdener, Zeynep Kaya, Turgay Dalkara

**Affiliations:** 1 Institute of Neurological Sciences and Psychiatry, Hacettepe University, Sıhhiye, Ankara, Turkey; 2 Institute of Neurological Sciences and Psychiatry, and Faculty of Medicine, Hacettepe University, Ankara, Turkey

## Abstract

**Background:**

Pain is generally concomitant with an inflammatory reaction at the site where the nociceptive fibers are activated. Rodent studies suggest that a sterile meningeal inflammatory signaling cascade may play a role in migraine headache as well. Experimental studies also suggest that a parenchymal inflammatory signaling cascade may report the non-homeostatic conditions in brain to the meninges to induce headache. However, how these signaling mechanisms function in patients is unclear and debated. Our aim is to discuss the role of inflammatory signaling in migraine pathophysiology in light of recent developments.

**Body:**

Rodent studies suggest that a sterile meningeal inflammatory reaction can be initiated by release of peptides from active trigeminocervical C-fibers and stimulation of resident macrophages and dendritic/mast cells. This inflammatory reaction might be needed for sustained stimulation and sensitization of meningeal nociceptors after initial activation along with ganglionic and central mechanisms. Most migraines likely have cerebral origin as suggested by prodromal neurologic symptoms. Based on rodent studies, a parenchymal inflammatory signaling cascade has been proposed as a potential mechanism linking cortical spreading depolarization (CSD) to meningeal nociception. A recent PET/MRI study using a sensitive inflammation marker showed the presence of meningeal inflammatory activity in migraine with aura patients over the occipital cortex generating the visual aura. These studies also suggest the presence of a parenchymal inflammatory activity, supporting the experimental findings. In rodents, parenchymal inflammatory signaling has also been shown to be activated by migraine triggers such as sleep deprivation without requiring a CSD because of the resultant transcriptional changes, predisposing to inadequate synaptic energy supply during intense excitatory transmission. Thus, it may be hypothesized that neuronal stress created by either CSD or synaptic activity-energy mismatch could both initiate a parenchymal inflammatory signaling cascade, propagating to the meninges, where it is converted to a lasting headache with or without aura.

**Conclusion:**

Experimental studies in animals and emerging imaging findings from patients warrant further research to gain deeper insight to the complex role of inflammatory signaling in headache generation in migraine.

## Background

Pain is generally concomitant with an inflammatory reaction of varying intensity at the site where the nociceptive fibers are activated. Migraine is probably no exception; there is ample experimental evidence, mostly from rodents, suggesting that the nociceptive trigeminocervical afferents mediating the headache can be activated by a sterile meningeal inflammatory process [1]. This process may directly originate within the meninges or be triggered by intrinsic brain activity such as cortical spreading depolarization (CSD) although debated [1–3]. Evidence from studies on mice and rats suggests that a parenchymal neuroinflammatory signaling between neurons, astrocytes and microglia, which eventually migrates to the meninges [2, 4–11], could be the potential link communicating a non-homeostatic event in the insensate brain to pain-sensitive meninges. Recent clinical imaging studies have provided supporting evidence for the presence of parenchymal as well as meningeal inflammation in migraine patients [12, 13]. In this review, our goal is to briefly summarize the current understanding of meningeal neurogenic inflammation and then focus on parenchymal inflammatory signaling in more detail. We should note from the beginning that these self-limited, physiological inflammatory responses should not be considered identical to conventional inflammation seen under pathological conditions despite shared mechanisms.

## Meningeal neurogenic inflammation and nociceptor activation

**Fig. 1:**
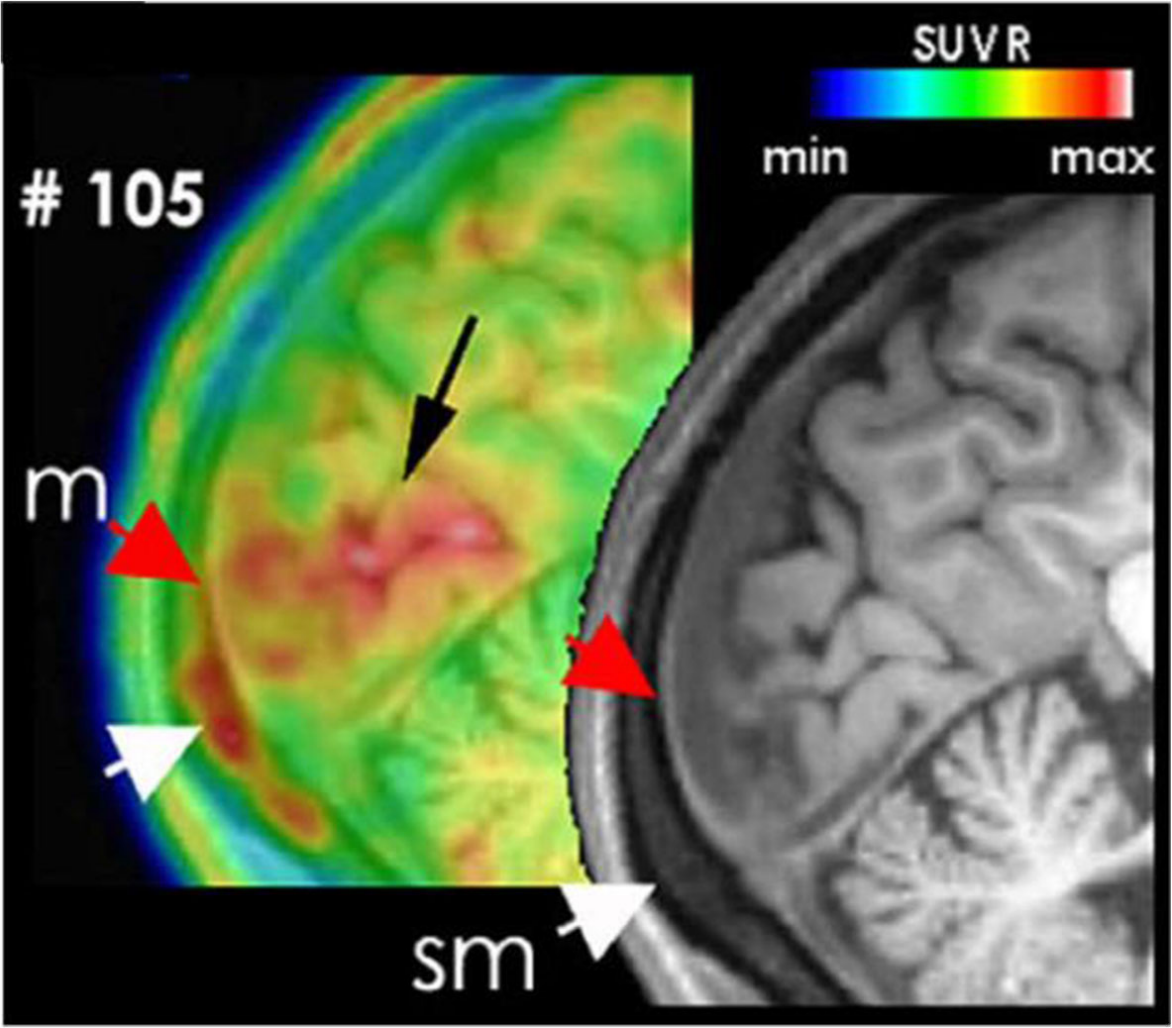
[(11) C]PBR28 PET/MRI showing inflammatory activity in the occipital cortex (black arrow), overlying meninges (red arrows), and bone marrow (white arrows) in a patient suffering from migraine with visual aura attacks (from [13] with permission)

Prolonged activation and sensitization of primary and central nociceptors within the trigeminocervical complex are thought to underlie the throbbing headache and allodynia during migraine. Rodent experiments suggest that a sterile meningeal inflammation initiated by release of peptides from trigeminocervical C-fibers and activation of resident inflammatory cells (mast cells, macrophages and dendritic cells) could contribute to sustained activation and sensitization of meningeal nociceptors [14–17]. Supporting these experimental findings, the association of meningeal inflammation with migraine headache has recently been shown by a positron emission tomography - magnetic resonance imaging (PET-MRI) study of 11 migraine patients using a highly sensitive inflammatory tracer [(11) C]PBR28 [13] (Fig. 1). Despite significant differences between species (e.g. the dominant peptide type expressed in nociceptors), it is likely that a form of subtle inflammatory reaction is required as a common mechanism to sustain stimulation of trigeminocervical nociceptive fibers for hours and even days following initial activation [16, 18]. Available evidence suggests that this inflammatory signaling may take place in the trigeminocervical ganglia as well as meninges and might involve central nociceptive pathways on chronification of headache [19, 20].

Meningeal nociceptive fibers can release a number of vasoactive peptides including calcitonin gene-related peptide (CGRP), pituitary adenylate cyclase-activating polypeptide (PACAP), substance P and neurokinin-A upon prolonged activation [1, 21]. By way of a monosynaptic reflex between the trigeminal nucleus caudalis and superior salivatory nucleus in brain stem, parasympathetic nerve fibers around meningeal vessels are concomitantly activated, releasing vasointestinal peptide (VIP), PACAP and acetylcholine [1, 22]. This parasympathetic reflex is thought to mediate the middle meningeal artery (MMA) dilation lasting about an hour in the wake of CSD [2, 23, 24]. Its short duration suggests that MMA dilation is not directly involved in headache but it still could be a measurable indicator of early trigeminovascular activation. This brief change in MMA diameter may have evaded detection in patients during spontaneous migraine attacks with MR angiograms, as patients were scanned several hours after attack onset [25, 26]. Indeed, MMA dilation in migraine has only been recorded at the initial phase of attacks induced by agents like CGRP or cilostazol [27, 28], which was significantly larger on the side ipsilateral to the pain unlike the dilations observed in intracranial arteries as a consequence of the direct vasodilatory actions of the agents used. In line with the view that MMA dilation may not be directly related to nociception, recent experimental studies have shown that systemically given CGRP antagonist, fremanezumab did not affect post-CSD MMA dilation and dural plasma protein extravasation (that has a parallel time course to MMA dilation) yet inhibited A∂-fiber mediated nociception [24, 29]. This is in contrast to action of triptans, which inhibit CGRP release from nociceptors, hence, suggests that triptans and CGRP antagonists may have different sites of actions on the bipolar trigeminocervical nerves (e.g. presynaptic varicosities or Ranvier nodes of peripheral meningeal branches or the ganglion) and dural inflammatory cells although they lead to similar pharmacological endpoints [19]. Among the peptide mediators released, CGRP is the one with the best documented role in migraine pathophysiology, because, CGRP concentration in plasma of jugular vein significantly increases during migraine attacks [30], intravenous CGRP infusion triggers migraine-like episodes in migraineurs [31] and anti-migraine treatments targeting either CGRP release (triptans) or CGRP peptide or its receptor (CGRP antagonists) are clinically effective [32].

**Fig. 2:**
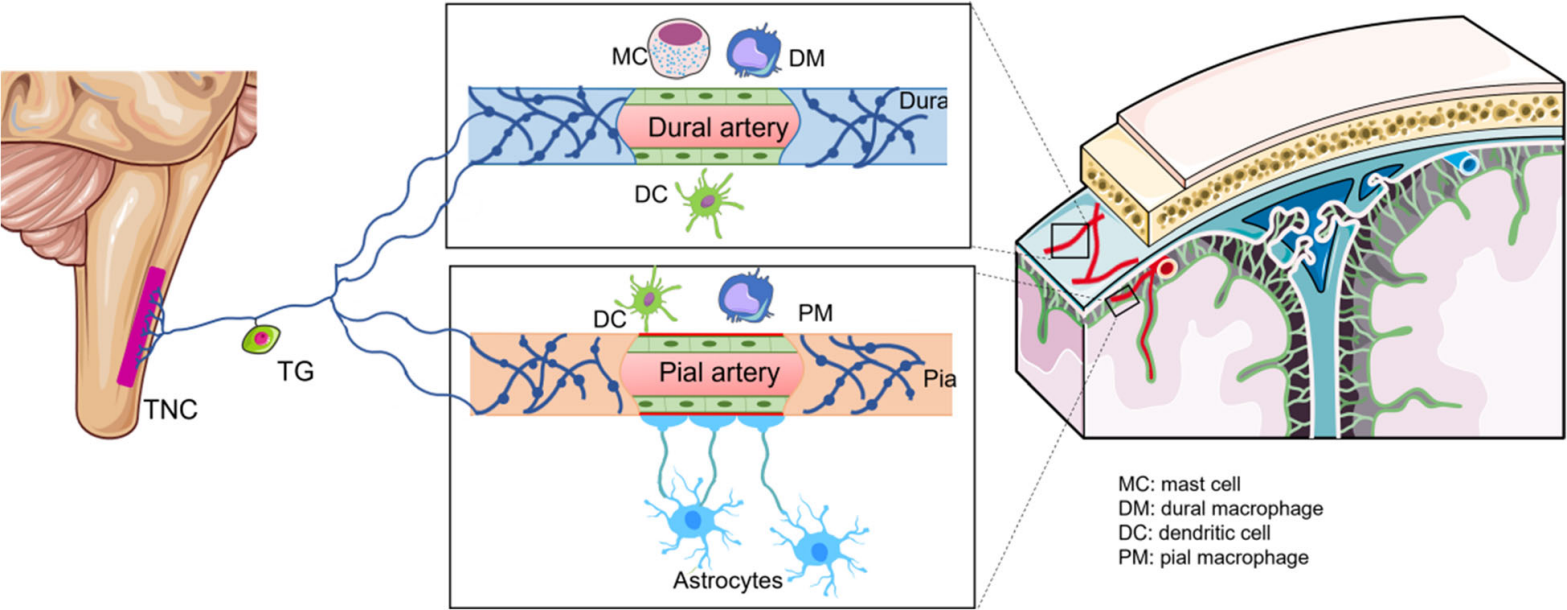
CSD activates trigeminal nociceptors around pial and dural vessels as well as meningeal macrophages and dendritic cells within 6–20 min in rodents [17]. Mast cell degranulation also contributes to the sterile meningeal inflammatory response. These inflammatory cells are in close proximity to the meningeal nociceptor fibers (pial nociceptive fibers and their axon collaterals around dural vessels) and may contribute to sustained stimulation and sensitization of nociceptors after initial activation, causing hours to days lasting headache. Blood-brain barrier (BBB) impermeable drugs such as anti-CGRP antibodies and sumatriptan can reach dural nociceptors located outside the BBB unlike pial nociceptors under the pial membrane. This figure is produced using Servier Medical Art (http://www.servier.com)

Several lines of experimental evidence suggest that activation of inflammatory cells such as mast cells, macrophages and dendritic cells contribute to meningeal neurogenic inflammation, at least in rodents [14–17] (Fig. 2). Dural macrophages activated by a single CSD reportedly retract their processes, assume a transient (20–50 min) circular phenotype and stay in close proximity to transient receptor potential cation channel subfamily (TRP) V member 1 (TRPV1)-positive meningeal nociceptors [17]. Dural mast cells are lined up along nociceptive fibers, suggesting a functional collaboration between them. Experiments in rodents have shown that mast cell activation contributes to activation of nociceptors by releasing several algesic mediators such as serotonin and prostaglandin I_2_ [33]. Tryptase is also secreted by mast cells, which activates the protease-activated receptors (PAR) found on dural afferents and causes migraine-like pain behaviors in mice [34–36]. Moreover, mast cells express purinergic receptors [37], which can be activated by adenosine triphosphate (ATP) elevated in the extracellular medium during inflammatory conditions. In mast cell-deficient animals, extracellular ATP-induced nociceptor firing is significantly weaker [38]. PACAP and glyceryl-trinitrate (GTN), both of which cause migraine-like headaches in humans are potent inducers of mast cell degranulation [39, 40]. VIP, Substance P, but not CGRP, degranulate human mast cells in vitro [41]. Although no CGRP receptor expression was detected in human mast cells [42], macrophages and dendritic cells [17] express CGRP receptors [43, 44] and can release mediators capable of activating nearby mast cells. The parasympathetic reflex can also contribute to mast cell activation by releasing PACAP and VIP. However, the evidence from humans for these inflammatory cellular reactions identified in experimental animals is yet incomplete. For instance, Khan et al., studied uptake of paramagnetic particles by macrophages in patients within 24 h of a migraine without aura (MO) attack [45], unfortunately however, their resolution allowed imaging macrophages only in the parenchyma, not in the meninges. Not surprisingly, they did not find increased macrophage activity in the pain processing hemisphere because the parenchymal neuroinflammatory signaling in migraine likely does not involve participation of inflammatory cells as discussed below.

As noted above, a strong support for involvement of vasoactive neuropeptides and sensory fibers in migraine pathogenesis is based on the success of anti-CGRP monoclonal antibodies, triptans and ditans in the clinic. CGRP antagonists inhibit CGRP or its receptors, whereas ergots and 5-hydroxytriptamine 1B/1D (5HT_1B/1D_) receptor agonist triptans, as well as the novel 5-HT_1F_ agonist lasmiditan suppress CGRP release from C-fibers [46–48]. Development of clinically useful triptans and CGRP antagonists is a striking example of how animal models of meningeal neurogenic inflammation provided information about neuropeptides that was instrumental and highly informative for clinical drug discovery. The successful relief of headache by monoclonal CGRP antibodies, which cannot permeate the blood-brain barrier (BBB) due to their large size, point to the significant role of CGRP in migraine nociception although the precise site of action is yet not clear [19]. Alleviation of migraine headache with sumatriptan that is also poorly BBB-permeable is in line with the effect of antibodies against CGRP and its receptor. These findings altogether suggest a site of action outside the BBB for triptans and CGRP antagonists either in the dura, the trigeminal ganglion or both. On the contrary, the clinical inefficiency of neurokinin-A antagonists that inhibit plasma protein extravasation in rodents led to the suggestion that the meningeal neurogenic inflammation might be a mere indicator of nociceptor activation but not the cause of headache [49–52]. It was further argued that the peripheral site of action of acute migraine drugs did not necessarily exclude the role of central mechanisms because a primarily intrinsic brain dysfunction could secondarily activate the peripheral trigeminal mechanisms.

Similarities (e.g. throbbing nature, photo and phonophobia) to headaches of subarachnoid hemorrhage or meningitis, known to be caused by meningeal irritation and inflammation, point to the meningeal nociception as the substrate of migraine headache as well [53]. However, despite the shared cascade of events, the intensity of inflammatory reaction is dramatically different between the two conditions. For example, while meningitis causes manifest gadolinium contrast enhancement in MRI, no enhancement has been detected during experimentally induced dural neurogenic inflammation [54–56] and there are is only a few case reports of contrast enhancement observed during severe familial hemiplegic migraine (FHM) attacks or prolonged aura [57–59]. The subtle inflammatory changes in meningeal neurogenic inflammation are therefore difficult to detect clinically by using routine neuroimaging methods. Indeed, a sensitive inflammatory tracer and sophisticated PET-MRI technology were necessary to be able to show this inflammatory activity in migraine patients suffering from frequent attacks [13].

## Origin of meningeal neurogenic inflammation

Activation and sensitization of trigeminocervical nociceptors, as part of an acute sterile inflammation, can take place at the level of meninges or the trigeminocervical ganglia, without any intrinsic brain involvement [60, 61]. Indeed, some migraine headaches may be initiated by direct activation of meningeal nociceptors with environmental irritants. For example, umbellulone evaporating from the leaves of headache tree (*Umbellularia californica*) is thought to stimulate TRP subfamily A member 1 (TRPA1) channels on meningeal nociceptors and thus cause headache [62]. Besides this direct mechanism of nociceptor activation, some irritants may act by inducing meningeal mast cell degranulation [15]. Another activator of the meningeal mast cells, GTN [39, 63] induces headache within 4–6 h after administration to migraineurs [64, 65] and meningeal neurogenic inflammation in rodents [63].

As noted above, a transient central dysfunction translated to headache by the trigeminocervical complex and its peripheral nociceptors are considered the most likely mechanism initiating migraine headache. A cerebral origin is indeed suggested by presence of several prodromal neurological symptoms preceding the headache [66]. Based on these, the central ‘migraine generator’ theory has been proposed [67–70]. PET studies of spontaneous or induced migraine attacks pointed to the brain stem areas including the periaqueductal gray matter and/or dorsolateral pons as potential trigger zones [67, 70–73]. However, considering that these areas cover several brain stem nuclei or neuronal populations having opposing roles in pain modulation, the resolution of the images was not sufficient to make a compelling argument for a pain-generating brain stem dysregulation [74, 75]. Imaging studies also disclosed an increased hypothalamic activity during migraine attacks, which led to suggestion of the hypothalamus as a potential trigger zone [76–78]. Indeed, an abnormal activity in hypothalamic nuclei and increased connectivity with thalamic and brain stem nuclei have been documented in the premonitory phase of migraine, as early as 24 h before symptomatic attack [78, 79]. This early hypothalamic activation is consistent with prodromal symptoms like appetite change, sleep abnormalities or autonomic alterations. However, the headache generator hypothesis currently lacks a convincing explanation for why a trigger zone should be present specifically for the trigeminal ophthalmic nociceptive system associated with migraine, whereas no such mechanism exists for neighboring areas transmitting/modulating the nociceptive impulses from rest of the body. Therefore, other intrinsic brain events arising in the parenchyma under suboptimal homeostatic conditions should also be considered for subsequent activation of peripheral and central nociceptors and, meningeal neurogenic inflammation.

In line with the latter view, although debated, migraine aura appears to be one of the triggers of the meningeal nociception [3, 16, 80, 81]. Several key experimental findings support this possibility: For example, it has been shown that CSD, the neurophysiological correlate of migraine aura [82], can lead to vasodilation, plasma extravasation and mast cell degranulation in dura about 20 min after a single CSD in rodents [2, 23, 24]. These changes can be prevented by trigeminal nerve denervation, indicating that they are caused by activation of the trigeminal nerve by CSD [23]. Indeed, both the trigeminal ganglion and second-order neurons in brain stem have been shown to start firing around 20 min after CSD in the rat [83–87]. Supporting animal experiments, headache also emerged within 35 min after the experimentally induced auras in migraineurs by hypoxia or by exercise (alone or combined with photic stimulation) or by photic stimulation [82, 88]. Focal hypoperfusion/hypoxia-induced headaches following an aura are one of the earliest symptoms of hereditary vasculopathies such as CADASIL [89, 90].

Conforming with the experimental findings, meningeal contrast enhancement was recorded with MRI during severe FHM or prolonged aura attacks [57–59] and, albumin leakage to dura was detected by single-photon emission computed tomography imaging during a migraine attack in a non-familial migraine case [91]. However, the most compelling evidence has recently been obtained by using a sensitive PET inflammation marker ([(11) C]PBR28), showing clear meningeal tracer uptake in 11 of 11 patients suffering from migraine with aura [13]. Importantly, the labeling was most prominent over the occipital cortex generating the visual aura and lasted several days after a migraine attack. The tracer uptake was strong enough to be visualized in single patients, creating the opportunity to test various hypotheses regarding the role of meningeal inflammation in migraine (e.g. whether it is suppressed by triptans and CGRP antagonists). Tracer uptake was extended into the overlying bone marrow, raising the interesting possibility that inflammatory bone marrow cells might also contribute to dural inflammation. This finding is also supported by the identification of direct vascular channels in rodents and humans, connecting skull bone marrow to the brain surface and meninges and allowing migration of myeloid cells [92].

About 2/3 of migraineurs, on the other hand, do not experience aura before headache (MO), which requires identification of brain dysfunctions other than CSD that can also lead to trigeminovascular activation. In fact, this need led to the hypothesis of a central dysfunction in pain pathways as discussed above. Alternatively, silent (asymptomatic) CSDs limited to gyri outside the visual and sensory cortices have been proposed to underlie MO attacks [93, 94]. The activation of parenchymal inflammatory signaling pathways and subsequent meningeal inflammation by cortical disturbances other than CSD has been proposed as another possibility [95]. According to this hypothesis, a mismatch between the rapidly escalating metabolic demand during intense glutamatergic transmission and synaptic energy supply can initiate the inflammatory signaling cascade and cause headache [96]. Migraine triggers such as fasting, sleep deprivation and hormonal changes may predispose to such a mismatch by impacting astrocytic energy supply mechanisms at transcriptional level [95–97]. For instance, sleep deprivation has been shown to lead to inadequate utilization of astrocytic glycogen because of the transcriptional changes induced that favor glycogen synthesis over glycogen breakdown [95, 96]. Glycosyl units liberated from glycogen are preferentially used over glucose for the uptake of extracellular potassium and glutamate by astrocyte processes due to the differential kinetics of enzymes involved and rates of glucose transport versus glycosyl liberation [95–97]. The same mechanism has also been shown to lower the CSD threshold in rodents by causing insufficient clearance of extracellular potassium and glutamate, hinting clues for emergence of migraine with and without aura attacks in the same individual by using a final common pathway [95–97]. Clinically supporting a potential role of metabolic perturbation in MO, headache emerged before as well as after the aura in migraineurs subjected to prolonged hypoxia [88]. The extensive literature about the potential role of a cerebral energy imbalance in migraine has recently been reviewed [98, 99].

## Parenchymal neuroinflammatory signaling

Several groups have reported a parenchymal inflammatory response to CSD, which might migrate to meninges and activate the trigeminovascular system and meningeal inflammation [2, 5–11]. Of note, this is simply a non-cellular, molecular inflammatory signaling cascade reporting the suboptimal homeostasis in brain but not an overt inflammation as seen, for example, in multiple sclerosis or around glial tumors. Such parenchymal signaling appears to be a prerequisite for migraine attacks starting within the brain except those initiated by direct activation of meningeal nociceptors. CSD-induced flux of algesic mediators such as H^+^, ATP and nitric oxide (NO) from interstitium to perivascular and subarachnoid spaces can activate perivascular pial nociceptors [23]. Indeed, in rats, firing of a group of neurons in trigeminal ganglion and nucleus caudalis concomitantly with CSD has been recorded, which can account for the auras coincident with headache [17, 83–87]. However, most of the nociceptive units started firing 15 min after a CSD wave when the tissue homeostasis had already been restored, in line with the clinical observation that headaches being delayed by 15–20 min after majority of migraine auras. This delay in dural nociceptor firing also conforms to the slowly rising MMA blood flow in the wake of CSD in rats and mice [2, 23, 24]. A 12–20 min delayed activation of dural macrophages and dendritic cells, following early activation of pial macrophages has also been proposed to explain this time lag after CSD [17].

**Fig. 3:**
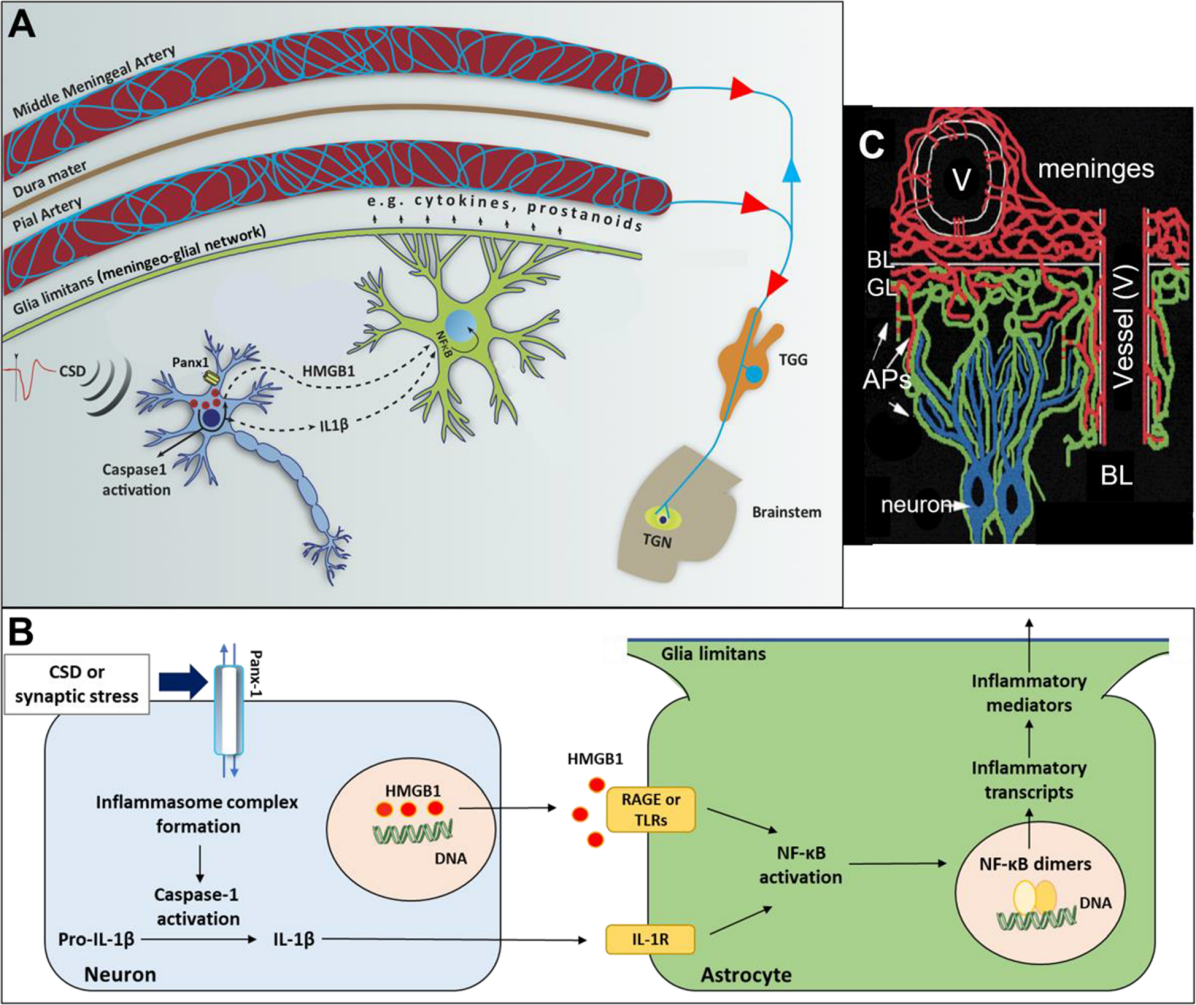
Neuronal Panx1 channel opening after CSD initiates a neuroinflammatory cascade, characterized by inflammasome formation and caspase-1 activation in neurons followed by release of HMGB1 and IL-1β, which trigger NF-κB nuclear translocation in astrocytes. **A**, **B** NF-κB leads to pro-inflammatory transcriptional activity and secretion of nitric oxide, cytokines and prostanoids from astrocyte endfeet, reaching meningeal nociceptors by way of meningo-astroglial network (**C**) to stimulate them (from [2 and 100], with permission). In A, red labels show agents used to inhibit each step in the inflammatory cascade and red circles represent propidium iodide (PI) influx through open Panx1 channels

Supporting the hypothesis of parenchymal inflammatory signaling, a recent PET/MRI study showed widespread uptake of an inflammatory tracer in the brains of 13 patients having frequent migraine with aura attacks [12]. According to the observations made in mice and rats, neural stress created by high extracellular potassium, glutamate and intracellular calcium as well as swelling during a single CSD leads to opening of pannexin 1 (Panx1) large-pore channels and release of interleukin-1β (IL-1β) and high mobility group box protein 1 (HMGB1) from neurons [2] (Fig. 3). These pro-inflammatory mediators cause nuclear translocation of the transcription factor nuclear factor-kappa B (NF-κB) in astrocytes and induce transcription of inflammatory enzymes and cytokines, which likely results in synthesis and release of prostanoids, cytokines, and NO from astrocytes to the perivascular and subarachnoid spaces [2]. The inflammatory mediators can directly reach nociceptive fibers around pial vessels over the glia limitans through a menigeo-astroglial network of interconnected cellular processes [100] (Fig. 3) or by way of cerebrospinal fluid (CSF) fluxes toward the surface in perivascular spaces. Axon collaterals from pial perivascular nociceptive fibers are thought to activate dural nociceptors and trigger the inflammatory signaling observed in rodent studies (Fig. 2). Indeed, an overt inflammatory tracer uptake in meninges overlying the occipital cortex was detected in the PET/MRI study of migraine with aura (MA) patients as mentioned above [13]. In the sections below, we will review the main elements of this parenchymal inflammatory cascade in more detail.

One advantage of the parenchymal inflammatory signaling hypothesis is that it can explain activation of meningeal nociceptors in the absence of CSD (in MO) as well as with CSD (in MA). For example, migraine triggers such as sleep deprivation or acute psychological stress induce transcriptional changes in astrocytes that direct glucose in astrocyte processes preferentially to glycogen synthesis, thereby, hindering glutamate and potassium clearance during rapidly escalating intense neuronal activity because glycogen-derived glycosyl units are essential to fuel astrocytic uptake mechanisms [95, 96]. This synaptic activity-related neuronal stress has been shown to initiate the parenchymal signaling cascade by opening of the neuronal Panx1 channels, causing caspase-1 activation and HMGB1 release in the absence of CSD in the mouse [95]. Thus, migraine triggers could potentially activate the parenchymal inflammatory signaling pathway and induce headache without needing CSD (i.e. aura).

Although multiple CSDs were previously proposed to be necessary for headache generation in rodents [101, 102], recent behavioral tests clearly showed that a single CSD evoked by noninvasive methods was sufficient to generate headache within an hour [103, 104] as suggested by previous electrophysiological recordings from dural afferents in rats [17, 83–87]. Of note, inflammatory response is expectedly heightened after multiple CSDs and is known to lead to activation of microglia, thus, can exhibit a more complex expression profile, which may be more akin to the inflammatory reaction in patients suffering from frequent migraine with aura attacks [9, 11, 105, 106]. Whereas multiple CSDs are useful as an experimental tool to disclose CSD-induced subtle changes, it should be kept in mind that, typically, a single CSD causes most of the auras in humans and, the associated hemodynamic changes matching the symptoms are localized to a single lobe or a few gyri, as captured with functional MRI or PET performed within 30 min of aura onset [82, 93, 107–112]. However, aura does not always lead to headache in patients, suggesting that, to be able to induce pain, CSD-induced inflammatory signaling has to coincide with a period when the somatosensory threshold is low. Fluctuations in the somatosensory threshold throughout the migraine cycle were previously shown with repeated functional MRIs (fMRI) during trigeminal stimulation and the extensive literature on migraine thresholds has recently been reviewed [113, 114]. Furthermore, pain threshold is well known to show inter and intra-individual variability and is modulated by several biological factors (e.g. genetic and hormonal). Indeed, some people never suffer from headaches at all [3].

## Neuronal stress sensors - Pannexin channels

Pannexins are membrane proteins harboring a large-pore ion channel. Although they are structurally similar to gap junction connexins, they assemble as membrane hemichannels without forming cell-to-cell coupled conduits. Of the 3 types of pannexin channels, Panx1 and Panx2 are present in the nervous system. Panx1 is widely expressed in both excitatory and inhibitory neurons as well as oligodendrocytes, astrocytes and microglia [115]. In neurons, it is mainly localized at the postsynaptic membrane [116] and functions as a modulator of glutamatergic transmission and a sensor for stressful conditions [117–119]. For example, when postsynaptic metabotropic N-methyl-D-aspartate (NMDA) receptors are activated, Src family of tyrosine kinases phosphorylate Panx1, allowing release of anandamide through Panx1 channel, which inhibits Ca^2+^ influx to the presynaptic terminal and glutamate release, hence, stabilizes excitatory activity [120]. When extracellular potassium and glutamate are high and NMDA receptors are intensely stimulated, Panx1 activation leads to inflammasome formation in neurons and IL-1β release, whereas more severe conditions jeopardizing cell survival cause caspase-mediated cleavage of C-terminal domain and persistent pore opening [121–123]. Panx1 activation by caspase-mediated cleavage releases large amounts of ATP to the extracellular medium, which recruits immune cells for clearance of apoptotic fragments [124].

Panx1 channels can be activated by various signals including increases in extracellular K^+^, glutamate and intracellular Ca^2+^ concentration [125], NMDA receptor stimulation [121], c-Jun N-terminal kinases, Src family of tyrosine kinases [126] and mechanical stress (e.g. spine swelling) [127]. Panx1 has two pore- opening states; a low-conductance ion channel with selective Cl^−^ permeability (no neuronal function is known) and a large-conductance nonselective channel permeable to molecules up to 900 Da, allowing considerable potassium and ATP efflux and, possibly, calcium influx [119, 128]. The latter feature is used to detect Panx1 opening with membrane-impermeant fluorescent dyes smaller than 900 Da like propidium iodide or YoPro-1 [124, 129]. This method has been instrumental to disclose the CSD-induced Panx1 activity in the brain [2, 10](Fig. 3). The exact mechanism of how neuronal Panx1 channels open in large-conductance state after CSD or synaptic metabolic stress is not entirely clear. High extracellular K^+^ and glutamate, NMDA receptor overactivation, high intracellular calcium, neuronal swelling may contribute to Panx1 large channel opening [2, 95]. It has recently been shown that CSD-induced stimulation of NR2A type NMDA receptor subunits activates Src-family kinases, which phosphorylate Y308 near the intracellular C-terminal, hence, promotes opening of Panx1 channels [10].

ATP released to the extracellular environment through the Panx1 large pore channels can activate nearby P2X7 receptors [130, 131]. Like Panx1 channels, P2X7 receptors are also able to switch to large conductance pore opening and activate inflammasome formation [130–132]. At least in macrophages, these two channels together are considered as a functional unit both capable of triggering formation of the inflammasome complex [125, 133, 134]. Whether or not neurons express P2X7 is controversial and the recent data do not support the expression of P2X7 in mature neurons in adult rodents [135–137]. Any close collaboration between neuronal Panx1 and P2X7 receptors on nearby astrocytes [138] and microglia processes [139] remains to be investigated.

Experimentally, it is possible to inhibit Panx1 or purinergic receptor activity with agents like carbenoxolone, probenecid, mefloquine ^10^panx peptide, disodium 4,4′-diisothiocyanatostilbene-2,2′-disulfonate or brilliant blue G [140]. Probenecid and mefloquine are clinically registered drugs [129], however, no reports have been published on their potential effect on migraine at the currently used doses although they are widely prescribed for gout and malaria treatment for many decades.

## Inflammasome complex formation and release of IL-1β and HMGB1 from neurons

Inflammasome complex formation is the downstream step to Panx1 channel activation. Inflammasomes are multimeric protein complexes that oligomerize in response to infectious agents or homeostatic disruptions. Inflammasome formation is a common step initiating most neuroinflammatory conditions by serving as a molecular machinery processing proinflammatory mediators to their active forms. An inflammasome complex is formed by clustering of the node-like receptors (NLRs), which detect the exogenous pathogen-associated molecular patterns (PAMPs) or endogenous damage-associated molecular patterns (DAMPs), and the adapter molecule, apoptosis-associated speck-like protein containing a C-terminal caspase recruitment domain (ASC) [141, 142]. Pro-caspase-1 dimerizes on the inflammasome complex and cleaves itself to release the active caspase-1, which mediates the proteolytic cleavage of pro-IL-1β in the cytoplasm to yield the active IL-1β. Inflammasome activation is also linked to HMGB1 translocation from nucleus to cytoplasm [143, 144]. Components of the inflammasome complexes are expressed in CNS cells [145, 146]. After CSD, inflammasome complex is rapidly formed as shown by appearance of the cleaved form of caspase-1 in neurons and detection of released IL-1β in CSF [2](Fig. 3). An increase in IL-1β expression as early as 10 min after a single noninvasively (optogenetically) triggered CSD has also been reported, which is followed by expression of several other pro-inflammatory genes [11]. However, the latter transcriptional changes detected in the cortical extracts may also reflect the NF-κB-induced transcriptional changes in astrocytes as discussed in the next section.

Parenchymal IL-1β production could also be a significant step leading to meningeal nociceptor activation in migraine without aura (i.e. without CSD) [95, 96]. Indeed, MO attacks are seen in patients with cyropyrin-associated periodic syndromes, in which IL-1β is overproduced due to mutations in the inflammasome component NLRP3. Interestingly, these migraine attacks are suppressed with IL1 receptor antagonist anakinra [147–149]. Further supporting a role for parenchymal inflammatory signaling in MO, IL-1β, prostaglandin E_2_, tumor necrosis factor-α (TNF-α), IL-6, and nitrite levels in the internal jugular vein (which drains mainly the brain parenchyma but not the meninges) were found to be elevated within the first hour of a MO attack [150–152].

HMGB1 is one of the non-histone proteins that binds to DNA in the nucleus and is expressed in high amounts by almost all cells [153]. It plays a role in chromosome stabilization, nucleosome mobility, DNA repair and control of transcription by binding to DNA [153]. Moreover, HMGB1 belongs to the family of alarmin proteins that initiate a rapid inflammatory response upon release from the cell [154]. HMGB1 passively leaks from necrotic or damaged cells but it can also be actively transported out of the cell after an inflammatory stimulus such as cell swelling, tissue injury or infection [155, 156]. In such cases, its three-dimensional structure changes by acetylation, phosphorylation or methylation of different amino acids [156]. This structural change allows the nuclear export signal to be exposed and leads to translocation of HMGB1 from nucleus to cytoplasm. HMGB1 can activate various inflammatory pathways including NF-κB in nearby cells bearing receptor for advanced glycation end products (RAGE) and toll-like receptors (TLRs) [156, 157].

About half of the neuronal nuclei lose their HMGB1 immunoreactivity right after a single CSD, whereas glial nuclei are not affected [2, 158] (Fig. 3). Single optogenetically-induced CSD also causes a comparable HMGB1 release to pinprick- or KCl-induced single CSDs (unpublished data), ensuring that HMGB1 release was caused by CSD but not experimental injury. The best available method to show CSD-induced HMGB1 release appears to be the immunohistochemistry (i.e. loss of nuclear HMGB1 immunoreactivity), whose specificity was validated by showing prevention of the CSD-induced NF-κB translocation in astrocytes with a neutralizing antibody against HMGB1 or by BoxA fragment of HMGB1 that shows antagonistic activity or by HMGB1-shRNA [2]. Some of the released HMGB1 leaks into CSF, however, it reaches detectable levels with Western blotting only after multiple CSDs. Furthermore, collecting CSF from small rodents with a stable intracerebroventricular cannula without injuring the brain is challenging. This small HMGB1 loss from brain tissue could also be detected in Western blots of cortex extracts as a decrease in HMGB1 levels 3 h after multiple, but not single, CSDs giving the impression that only multiple CSDs could cause HMGB1 release [5]. After a single CSD induced by either pinprick or KCl or optogenetically, NF-κB rapidly translocates to the nucleus (i.e. becomes activated) in astrocytes in response to HMGB1 released from neurons in the mouse brain [2, 158–160] (Fig. 3).

HMGB1 has also been linked to several other pain-associated conditions. HMGB1 translocation in spinal cord neurons contributes to bone cancer-related hyperalgesia [161]. HMGB1 can directly induce pain, as shown by subcutaneous injection of HMGB1 to the paw or application over the sciatic nerve [162, 163]. HMGB1 expression is reportedly increased in the spinal cord in diabetic neuropathic pain [164] and mechanical compression pain, in which anti-HMGB1 neutralizing antibodies reverse the pain-related behavior in rodents [165].

## Astrocyte and microglia activation and NF-κB pathway

Release of IL-1β and HMGB1 activates the NF-κB pathway in neighboring cells. Transcriptional NF-κB activity in astrocytes and microglia is particularly important for orchestrating the neuroinflammatory response; however, the timing and pro or anti-inflammatory nature of transcriptional activity may vary between astrocytes and microglia. The NF-κB transcription factor family operates by combining five subunits in pairs [166, 167]. These subunits are p65, cRel, RelB, p52, and p50. Each subunit contains a region called Rel homology domain [166]. This region contains the specific amino acid sequence that allows the subunit to form a pair, enter the cell nucleus and bind to DNA. The transactivation zone necessary for transcription is found only in the p65, cRel, and RelB subunits. Therefore, when pairs that do not contain one of these subunits (e.g. p50/p50) are attached to DNA, they only modify activities of the transcription-inducing pairs [166]. The NF-κB signaling operates through two alternative pathways, classical and non-classical. While the classical pathway plays a role in innate immunity and cell survival, the non-classical pathway is effective in acquired immune response. In conditions that create a sterile immune response such as inflammatory signaling induced by CSD, the classical pathway is activated [2, 9]. Effective subunits in this pathway are p65, p50 and cRel [168].

Pro-inflammatory mediators released from neurons or astrocytes activate microglia, the main immune cells of the CNS. After multiple CSDs, overt microglial activation is delayed by 24 h and depends on TLR2/4 [169]. These microglia are hypertrophic, exhibit increased motility/migration and phagocytic activity, reactive oxygen species production and IL-1β and TNF-α secretion [105, 106, 170–173]. Importantly, these changes are observed only after multiple CSDs but not a single CSD. The delayed emergence of inflammatory changes after the induction of CSD suggests that activated microglia may function in elimination or repair of the injured (swollen) dendritic spines during repeated CSDs [106, 174, 175]. Interestingly, naïve FHM mutant mice show microglial activation characterized with increased branching even under baseline conditions [176]. This may be caused by the recurrent spontaneous CSDs in these animals. Moreover, microglia may also play a downregulatory role in resolution of the parenchymal inflammatory signaling by switching to an anti-inflammatory phenotype, however, this remains to be investigated [177].

## Clinical outlook and conclusions

A fundamental step to further our understanding on migraine pathophysiology is to document the presence of the above-discussed mechanisms in migraine patients. Recent advances in neuroimaging techniques are promising in this regard. Detection of the meningeal inflammation over the occipital cortex exhibiting parenchymal inflammatory tracer uptake in patients suffering from migraine with visual aura is particularly encouraging, although preliminary [13] (Fig. 1). Improved tracers, if successful, can be instrumental to resolve some of the controversies on the role of meningeal inflammation in migraine. In this study, researchers took advantage of a sensitive PET ligand, [(11) C]PBR28 that detects the inflammatory activity in astrocytes and microglia by binding to the translocator protein (TSPO) located on outer mitochondrial membranes of active inflammatory cells. [(11) C]PBR28 was able to mark both CNS and meningeal involvement, as TSPO is upregulated in activated parenchymal glial cells as well as activated macrophages and peripheral immune cells. These observations are translationally relevant because experimental studies using the same radioligand showed increased uptake in the ipsilateral rat brain for up to 15 days after multiple CSDs [178]. Of note, the activity of the glia and meningeal inflammatory cells visualized by [(11) C]PBR28 uptake might not solely be caused by a pro-inflammatory state but might also involve anti-inflammatory activity because majority of the patients had their last headache attack a few days before imaging. Indeed, ongoing studies in our laboratory show that resolution of the inflammatory activity induced by a single CSD takes at least 3 days in the mouse brain [159, 160].

[(11) C]PBR28 PET may also provide insight into the relationship between inflammatory signaling and headache in secondary headache disorders. As in migraine, parenchymal inflammation can also play a role in post-seizure headache, as suggested by studies showing activation of the neuroinflammatory cascade in seizure models [179, 180]. Similarly, acute ischemia attacks are associated with headache [181]; but not always, possibly because ischemic nociceptive fibers around pial vessels become dysfunctional due to hypoperfusion of these arteries (the cause of the stroke) and, hence, cannot fire despite inflammatory mediators reaching to the pia from the ischemic brain. Indeed, headache is more prevalent after intracerebral hemorrhages and subarachnoid hemorrhage, which also activate Panx1 and downstream pathways [182].

In conclusion, experimental studies in animals and emerging imaging findings from patients warrant further research to gain deeper insight into the complex role of inflammatory signaling in headache generation. Research over the past 50 years have revolutionized our understanding of migraine, however, many unanswered questions and controversies remain. We need cutting-edge tools to directly and comprehensively study the complex nociceptive mechanisms in experimental animals and also high-resolution advanced imaging technologies to assess the significance of basic findings in the clinic. Admittedly, current experimental and clinical methods have shortcomings to provide unequivocal evidence for competing hypotheses.

## Data Availability

Not applicable.

## Abbreviations

TSPO: 18kD translocator protein
BBB: Blood-brain barrier
CADASIL: Cerebral autosomal dominant arteriopathy with subcortical infarcts and leukoencephalopathy
CGRP: Calcitonin gene-related peptide
CSF: Cerebrospinal fluid
CSD: Cortical spreading depolarization
DAMP: Damage-associated molecular pattern
FHM: Familial hemiplegic migraine
GTN: Glyceryl-trinitrate
HMGB1: High mobility group box protein 1
IL: Interleukin
MMA: Middle meningeal artery
MO: Migraine without aura
MA: Migraine with aura
NO: Nitric oxide
NMDA: N-methyl-D-aspartate
NLR: Node-like receptor
NF-κB: Nuclear factor-kappa B
Panx: Pannexin
PAMP: Pathogen-associated molecular pattern
PACAP: Pituitary adenylate cyclase-activating polypeptide
PET/MRI: Positron emission tomography/magnetic resonance imaging
KCl: Potassium chloride
PAR: Protease-activated receptor
RAGE: Receptor for advanced glycation end products
TLR: Toll-like receptor
TRP: Transient receptor potential cation channel subfamily
TNF: Tumor necrosis factor
VIP: Vasoactive intestinal polypeptide
